# RETRACTED: Majid et al. An Extensive Pharmacological Evaluation of New Anti-Cancer Triterpenoid (Nummularic Acid) from *Ipomoea batatas* through In Vitro, In Silico, and In Vivo Studies. *Molecules* 2022, *27*, 2474

**DOI:** 10.3390/molecules30112330

**Published:** 2025-05-27

**Authors:** Muhammad Majid, Anam Farhan, Muhammad Imran Asad, Muhammad Rashid Khan, Syed Shams ul Hassan, Ihsan-ul Haq, Simona Bungau

**Affiliations:** 1 Faculty of Pharmacy, Capital University of Science and Technology, Islamabad 44000, Pakistan; majidpharma808@gmail.com; 2 Department of Biology, School of Science and Engineering, Lahore University of Management Sciences, Lahore 54810, Pakistan; anam.farhan764@gmail.com; 3 Department of Pharmacy, Faculty of Biological Sciences, Quaid-i-Azam University, Islamabad 44000, Pakistan; miasad@bs.qau.edu.pk; 4 Department of Biochemistry, Faculty of Biological Sciences, Quaid-i-Azam University, Islamabad 44000, Pakistan; mrkhanqau@yahoo.com; 5 Shanghai Key Laboratory for Molecular Engineering of Chiral Drugs, School of Pharmacy, Shanghai Jiao Tong University, Shanghai 200240, China; 6 Department of Natural Product Chemistry, School of Pharmacy, Shanghai Jiao Tong University, Shanghai 200240, China; 7 Department of Pharmacy, Faculty of Medicine and Pharmacy, University of Oradea, 410028 Oradea, Romania

The journal retracts the article “An Extensive Pharmacological Evaluation of New Anti-Cancer Triterpenoid (Nummularic Acid) from *Ipomoea batatas* through In Vitro, In Silico, and In Vivo Studies” [1] cited above.

Following publication, concerns were brought to the attention of the publisher regarding a element duplications contained with images presented in this article [1].

Adhering to our standard procedure, an investigation was conducted by the Editorial Office and Editorial Board that confirmed highly similar elements between the panels “Control—12 h” and “NA—12 h” presented in Figure 3A. While the authors cooperated within the investigation, they were unable to satisfactorily explain the overlap nor provide material deemed satisfactory by the Editorial Board to verify the integrity of the images. As a result, the Editorial Board has lost confidence in the integrity of the findings and decided to retract this publication [1], as per MDPI’s retraction policy (https://www.mdpi.com/ethics#_bookmark30).

This retraction was approved by the Editor-in-Chief of the journal *Molecules*.

The corresponding authors did not agree to this retraction. The remaining authors did not provide a comment on this decision.

## References

[1] Majid M., Farhan A., Asad M.I., Khan M.R., Hassan S.S.u., Haq I.-u., Bungau S. (2022). RETRACTED: An Extensive Pharmacological Evaluation of New Anti-Cancer Triterpenoid (Nummularic Acid) from *Ipomoea batatas* through In Vitro, In Silico, and In Vivo Studies. Molecules.

